# Aphthous-Like Lesions in a Patient Across Two Courses of COVID-19 Infection: A Case Report

**DOI:** 10.7759/cureus.72552

**Published:** 2024-10-28

**Authors:** Irfan Corovic, Bojana Simovic Markovic, Mladen M Maksic, Dusan Radojevic, Isidora Stanisavljevic, Selma Habibović, Emina Ćorović Ličina, Irfan Sabotic, Lejla Suljic, Marina Jovanovic

**Affiliations:** 1 Center for Molecular Medicine and Stem Cell Research, Faculty of Medical Sciences, University of Kragujevac, Kragujevac, SRB; 2 Department of Internal Medicine, General Hospital of Novi Pazar, Novi Pazar, SRB; 3 Department of Internal Medicine, Faculty of Medical Sciences, University of Kragujevac, Kragujevac, SRB; 4 Department of Urology, General Hospital of Novi Pazar, Novi Pazar, SRB; 5 Department of Otorhinolaryngology, Faculty of Medical Sciences, University of Kragujevac, Kragujevac, SRB

**Keywords:** aphthous ulcer, covid 19, oral diseases, oral mucosal lesions, sars-cov-2

## Abstract

Numerous changes in the oral cavity have been associated with COVID-19 infection, including the appearance of aphthous-like lesions. However, the precise relationship between COVID-19 and aphthous-like lesions remains unclear and poorly explained. We present the case of a 34-year-old man with no prior medical conditions who developed aphthous-like lesions three days after the onset of the COVID-19 infection. Remarkably, the patient had experienced similar aphthous-like lesions two years earlier, also within the first three days of COVID-19 infection. He had no history of aphthous-like lesions before or between these two mild courses of COVID-19 infection. The patient was treated with corticosteroids and antiseptics, resulting in the complete resolution of the oral lesions. Given the limited evidence currently available, this case report highlights a potential direct association between COVID-19 infection and the development of aphthous-like lesions.

## Introduction

Severe acute respiratory syndrome coronavirus 2 (SARS-CoV-2) is the cause of the COVID-19 infection, a highly contagious disease that emerged at the end of 2019 in Wuhan, China. In March 2020, the World Health Organisation declared the COVID-19 outbreak a pandemic, and since its beginning, it has been responsible for over six million deaths as of May 2024 [1,2]. COVID-19 is primarily a lung disease, but it can cause numerous systemic manifestations, including changes in the oral cavity [1,3]. The main structural feature of the SARS-CoV-2 virus is the presence of a spike protein, which enables binding to human cells via the transmembrane angiotensin-converting enzyme 2 (ACE2) receptor, located in the lungs, kidneys, esophagus, ileum, colon, myocardium, and the epithelium of the oral cavity [1,3,4]. The localization of the ACE2 receptor in the oral cavity, as well as the presence of the virus in the saliva of infected individuals, indicates that COVID-19 infection may be a direct cause of the development of oral lesions [3,4]. However, the exact nature of the relationship and the underlying factors that associate COVID-19 infection with the development of oral lesions are still largely unclear.

Therefore, this case report aims to identify the direct relationship between COVID-19 infection and the development of oral lesions by presenting a unique case of aphthous-like lesions occurring during two separate episodes of COVID-19 infection in the same patient. This report will offer new insights into the understanding of COVID-19's oral manifestations and explore the mechanisms involved in this phenomenon.

## Case presentation

**Figure 1:**
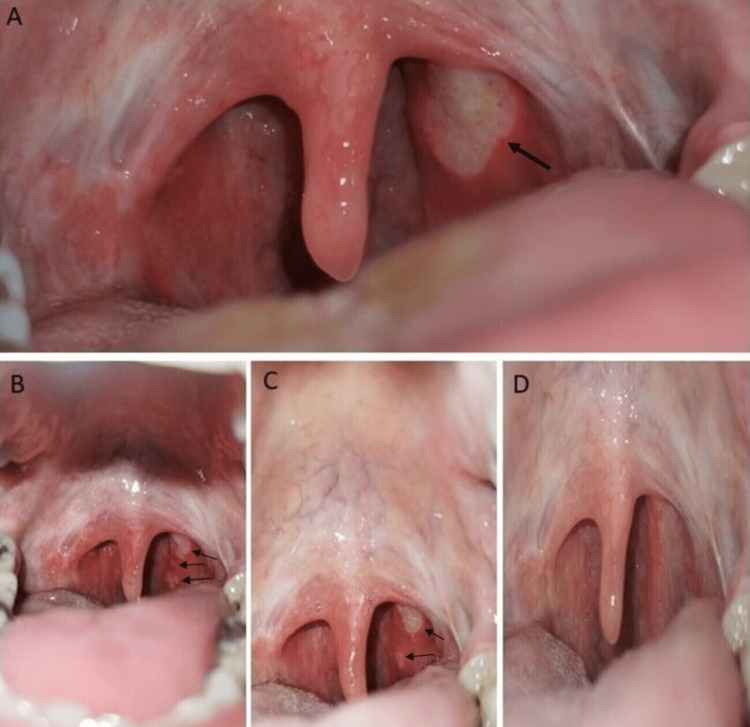
A: Aphthous-like lesions (black arrow) during the first COVID-19 episode two years ago; B: Aphthous-like lesions on Day 2; C: Aphthous-like lesions on Day 7; D: Complete resolution of the aphthous-like lesions on Day 20

A 35-year-old man presented to our outpatient clinic with the following signs and symptoms: fever, malaise, dry cough, throat pain, and odynophagia. The patient was in his usual state of health until approximately two days before evaluation (Day 0), when he reportedly developed a fever of up to 37.7 °C and malaise. On Day 1, a dry cough and mild throat pain started, and on the next day (Day 2), the throat pain worsened with the development of significant odynophagia, prompting the patient to visit our outpatient clinic for evaluation. During these two days, he did not take any medications, including over-the-counter drugs or supplements. The patient had no history of chronic diseases, drug allergies, tobacco or electronic cigarette use, alcohol consumption, or illicit drug use. The patient had a history of close contact with a COVID-19 patient, as his wife tested positive for COVID-19 via an antigen test one week before his evaluation in our outpatient clinic. She exhibited mild symptoms, including a sore throat and dry cough but showed no changes in the oral cavity. Two years ago, the patient himself tested positive for COVID-19 through an antigen test, during which he experienced mild symptoms, such as a sore throat, malaise, and fever, for which he used only acetaminophen. Three days after the onset of that infection, he developed aphthous-like oral lesions on the posterior pharyngeal wall. The oral lesions resolved concurrently with the COVID-19 infection, alongside the application of a 3% hydrogen peroxide solution three times a day and a 0.1% triamcinolone acetonide mouthwash solution three times a day. During the evaluation, the patient provided photographs from his mobile phone that depicted these oral changes (Figure 1A). He denied having any oral lesions before and after the mentioned COVID-19 infection. His family history included hypertension in his father.

He tested positive for SARS-CoV-2 using a nasal swab for rapid antigen testing (Abbott Panbio™, Abbott Laboratories, Green Oaks, Illinois, United States). During the examination, the patient’s vital signs were within normal limits. The intraoral assessment revealed multiple aphthous-like lesions on the posterior pharyngeal wall, with sizes ranging from 2 to 10 mm and shapes varying from round to irregular (Figure 1B). The remainder of the physical examination was unremarkable. The C-reactive protein level was 12.4 mg/l (the reference range is less than 5.0 mg/l), and the remainder of the laboratory test results were within the reference range. We performed several other diagnostic tests to exclude secondary causes of aphthous-like lesions, all of which were negative (Table 1). Chest radiography was performed, and it was normal.

**Table 1:** Laboratory data

Variable	Day 2	Day 20	Reference Range, Adults
Erythrocyte sedimentation rate (ESR)	5 mm/h	/	0-20 mm/h
Thyroid-stimulating hormone (TSH)	1.46 mIU/l	/	0.27-4.2 mIU/l
Free thyroxine (FT4)	17.0 pmol/l	/	11.5-22 pmol/l
Ferritin	73.64	/	21.8-274.7 ng/ml
Serum iron	10.3 µmol/l	/	6.3-31.3 µmol/l
Vitamin B12	506.2 pmol/l	/	145-569 pmol/l
Antinuclear antibodies (ANA)	0.6	/	0-1.2
Transglutaminase (tTG) immunoglobulin (Ig) A	0.6 U/ml	/	0-10 U/ml
tTG IgG	1.1 U/ml	/	0-10 U/ml
Fecal calprotectin	negative	/	negative
Fecal Helicobacter pylori antigen	negative	/	negative
Human immunodeficiency virus (HIV) antibody/antigen	0.08 s/co	/	< 1 s/co
Hepatitis B surface (HBs) antigen	0.00 s/co	/	< 0.9 s/co
Hepatitis C virus (HCV) antibody	0.03 s/co	/	< 0.9 s/co
Varicella-zoster virus (VZV) IgM	0.73 U/ml	0.62 U/ml	< 10 U/ml
VZV IgG	0.21 U/ml	0.14 U/ml	< 25 U/ml
Cytomegalovirus (CMV) IgM	2.33 U/ml	1.23 U/ml	< 10 U/ml
CMV IgG	0.21 U/ml	0.11 U/ml	< 25 U/ml
Epstein-Barr virus (EBV) viral capsid antigen (VCA) IgM	1.99 U/ml	0.99 U/ml	< 9 U/ml
EBV IgG	0.11 U/ml	0.21 U/ml	< 10 U/ml
Herpes simplex virus (HSV) type 1 IgM	0.24 U/ml	0.14 U/ml	< 20 U/ml
HSV type 1 IgG	0.13 U/ml	0.21 U/ml	< 25 U/ml
HSV type 2 IgM	0.45 U/ml	0.35 U/ml	< 10 U/ml
HSV type 2 IgG	0.76 U/ml	0.35 U/ml	< 25 U/ml
Oral cavity swab	negative	/	negative

The patient was prescribed chlorhexidine 0.12% mouthwash solution five times per day, triamcinolone acetonide 0.1% mouthwash solution three times per day, and oral acetaminophen as needed for high fever, along with home isolation measures. The next follow-up evaluation was scheduled for five days later or sooner if the condition deteriorated. The patient returned for the scheduled evaluation on Day 7 with significant improvement in symptoms; he had had no fever for the past three days without using acetaminophen, and the cough had stopped. Additionally, throat pain and odynophagia had improved significantly, and the oral lesions were slightly regressing (Figure 1C). The C-reactive protein level had decreased to 4.3 mg/l. Other laboratory test results and the follow-up chest radiography were normal. He was given instructions to continue the prescribed therapy for the next five days and to complete home isolation measures before returning to his normal activities. Approximately two weeks later (Day 20), he appeared free of symptoms with a complete resolution of oral lesions (Figure 1D).

## Discussion

Aphthous-like lesions, painful spots on the oral mucosa, are characterized by an erythematous halo and are covered with a fibrous layer. They have a global prevalence ranging from 5% to 25% and frequently recur. These lesions predominantly appear on the non-keratinized mucosal areas of the lips, cheeks, and tongue, and occasionally on the keratinized mucosal regions of the gums and palate [5]. They can be categorized as idiopathic when referred to as recurrent aphthous stomatitis or secondary when attributed to factors such as stress, viral and bacterial infections, medications, local trauma, nutritional deficiencies, and autoimmune diseases like Behçet's disease, coeliac disease, systemic lupus erythematosus, and Crohn's disease [6].

The precise mechanism underlying the formation of aphthous lesions remains incompletely understood, but it is believed that local and/or systemic immune response dysregulation, specifically an overactivity of the Th1 immune response along with increased synthesis of pro-inflammatory cytokines, such as tumor necrosis factor-alpha (TNF-α), interleukin-2 (IL-2), and interferon‐gamma (IFN-γ), as well as decreased secretion of anti-inflammatory cytokines, such as IL-10, plays a fundamental role in the onset of this condition [5]. Additionally, a decreased CD4/CD8 ratio and a reduced number of regulatory T cells are among other proposed immune mechanisms for the development of these lesions [1]. Notably, one of the most common oral manifestations observed during SARS-CoV-2 infection is the occurrence of aphthous-like lesions [7], as observed in our patient.

The relationship between SARS-CoV-2 and the development of aphthous-like lesions remains poorly understood [7]. The SARS-CoV-2 virus gains entry and replicates within the epithelial cells of both the oral mucosa and salivary glands via ACE2 and serine protease TMPRSS receptors. This process leads to the destruction of the epithelial cells and initiates an inflammatory response. These findings strongly imply that SARS-CoV-2 may directly contribute to the emergence of various oral manifestations, including aphthous-like lesions [8]. Since the oral mucosa can serve as the initial site of SARS-CoV-2 infection, it can be the source from which the virus further spreads to the lungs. Moreover, preliminary clinical studies have indicated that using mouthwash with antiseptics and addressing oral mucosal lesions may potentially result in a reduction in the severity of SARS-CoV-2 infection [7,8]. Other potential mechanisms for the onset of these lesions during SARS-CoV-2 infection include immune system dysregulation, characterized by increased local and systemic production of pro-inflammatory cytokines, such as TNF-α and IL-1β [8], as well as the potential impact of medications used in treating SARS-CoV-2 infection [7]. Additionally, poor oral hygiene and trauma during intubation may serve as significant contributing factors to the development of the aforementioned changes in the oral mucosa. Furthermore, SARS-CoV-2 can induce alterations in the microbiota and potentially influence the formation of oral lesions [7]. It is also important to note that the occurrence of aphthous lesions is associated with a milder form of COVID-19, with the percentage of aphthous lesions decreasing from 25% to 7% in comparison to the group with a moderate form of the disease, for reasons that are currently unknown [7].

Given that a comprehensive diagnostic assessment excluded most potential secondary causes for the changes observed in the oral cavity of our patient, and considering the remarkable alignment of the clinical course and recovery process with the resolution of COVID-19 infection in two disease courses, it is reasonable to empirically conclude that SARS-CoV-2 infection is the primary causative factor behind the appearance of aphthous-like ulcers in our patient. Significantly, this case represents, to the best of our knowledge, the first documented case of aphthous-like ulcers manifesting exclusively during two distinct episodes of COVID-19 infection in the same patient over a two-year period, providing strong support for our assertions.

A limitation of this case report, as well as of most studies on this pathology, is the lack of pathohistological and molecular analyses of changes in the oral cavity. Given the extensive impact of the COVID-19 pandemic on the global population and its serious consequences, along with the largely unknown long-term effects of COVID-19 infection, particularly on the immune system and autoimmune phenomena [9], further research on this pathology should consider this to fully understand the pathogenesis of COVID-19.

## Conclusions

Aphthous-like ulcers are prevalent and often observed in patients with COVID-19, either as a direct consequence of the virus or due to immunological disruptions. Oral lesions could be the first sign of COVID-19 infection, and their timely diagnosis and treatment can possibly influence the course of the disease. Further studies are needed to elucidate the molecular mechanisms underlying the connection between COVID-19 infection and oral manifestations of the disease.
